# Authentic SP-based teaching in spite of COVID-19 – is that possible?

**DOI:** 10.3205/zma001417

**Published:** 2021-01-28

**Authors:** Lilly Hartmann, Jens J. Kaden, Renate Strohmer

**Affiliations:** 1 Universität Heidelberg – Medizinische Fakultät Mannheim, Mannheim, Germany

**Keywords:** teaching, education, communication, patient simulation, feedback, physician-patient relations

## Abstract

**Objective: **Medical conversation plays a central role in disease management and therapy. In teaching, standardized patients (SPs) are increasingly being used to present conversation situations with students and provide feedback afterwards. In order to maintain this teaching concept under pandemic conditions, a digital model was developed that should offer both high security and high authenticity.

**Methodology: **A total of 176 teaching units of 45 minutes each were conducted digitally from May to August 2020. During the teaching units, medical students conducted interviews with SPs portraying various patients. The online conference software “HeiConf” was used for this purpose. During the teaching units, a total of 354 students were able to practice conversation techniques such as NURSE and SPIKES. After the teaching units, feedback was provided by students and SPs.

**Results: **The digital lessons about medical conversation with SPs received positive feedback from SPs and students. The authenticity of the role portrayal of SPs seemed to be unaffected by the new format. Students were successful in training and observing conversation techniques. However, aspects of non-verbal communication, atmosphere and group dynamics as well as further discussions could not be carried out to the same extent as in the usual classroom teaching.

**Conclusion: **The conversion of SP-based teaching to a digital format was successful in a short period of time and was able to prevent a cancellation of teaching units about medical conversation. Concrete conversation techniques could be tried out digitally by students. Due to the deficits of digital teaching in terms of non-verbal communication and atmosphere, a blended-learning format is planned for the future. In the first instance, concrete conversation techniques will be learned online in order to focus more on profound aspects of communication and discussions in a later physical teaching unit with SPs, thus enabling a learning experience that is as authentic as possible.

## Zusammenfassung

**Zielsetzung: **Die ärztliche Gesprächsführung spielt eine zentrale Rolle bei der Krankheitsbewältigung und Therapie. In der Lehre kommen immer häufiger Simulationspersonen (SPs) zum Einsatz, die mit den Studierenden Gesprächssituationen darstellen und im Anschluss Feedback geben. Um dieses Unterrichtskonzept auch unter Pandemiebedingungen aufrechtzuerhalten, wurde ein digitales Modell entwickelt, das sowohl hohe Sicherheit, als auch hohe Authentizität bieten sollte.

**Methodik: **Insgesamt wurden von Mai bis August 2020 176 Unterrichtseinheiten à 45 Minuten digital durchgeführt. Im Rahmen der Unterrichtseinheiten führten Medizinstudierende Gespräche mit SPs, die verschiedene PatientInnen darstellten. Hierbei wurde die Online-Konferenz-Software „HeiConf“ genutzt. Im Rahmen der Unterrichtseinheiten konnten insgesamt 354 Studierenden Gesprächstechniken wie NURSE und SPIKES üben. Im Anschluss an die Unterrichtseinheiten wurde eine Rückmeldung von Studierenden und SPs eingeholt.

**Ergebnisse: **Der digitale Unterricht zu ärztlicher Gesprächsführung mit SPs erhielt positive Rückmeldungen seitens der SPs und Studierenden. Die Authentizität der Rollendarstellung der SPs erschien von dem neuen Format unbeeinträchtigt. Das Trainieren und Beobachten von Gesprächstechniken gelang Studierenden gut. Aspekte der nonverbalen Kommunikation, Atmosphäre und Gruppendynamik sowie weiterführende Diskussionen konnten jedoch nicht in dem Umfang aufgegriffen werden, wie dies bei der üblichen Präsenzlehre geschieht.

**Schlussfolgerung: **Die Umstellung der SP-basierten Lehre auf ein digitales Format ist in kurzer Zeit gelungen und konnte einen Ausfall des Unterrichts zu ärztlicher Gesprächsführung verhindern. Konkrete Gesprächstechniken konnten digital von Studierenden erprobt werden. Aufgrund der Defizite von digitalem Unterricht in Bezug auf nonverbale Kommunikation und Atmosphäre wird für die Zukunft ein Blended-Learning-Format angestrebt. Hierbei sollen zunächst konkrete Gesprächstechniken online erlernt werden, um in einer späteren physischen Unterrichtseinheit mit SPs den Fokus stärker auf tiefgreifende Aspekte der Kommunikation und Diskussionen zu legen und so eine möglichst authentische Lernerfahrung zu ermöglichen.

## 1. Introduction

It is now widely known that communication between doctors and patients plays a decisive role in healing processes and diagnostics [1], [2]. At the Mannheim Medical Faculty, medical communication has therefore been anchored in the curriculum since 2006. Part of the teaching of medical conversation is carried out with standardized patients (SPs) who portray different patient scenarios. The effectiveness of SP-based teaching has been empirically confirmed [3], [4]. In SP teaching, students can train their communication skills and conversation techniques such as NURSE and SPIKES [5], [6]. In the Mannheim learning environment TheSiMa [https://www.umm.uni-heidelberg.de/studium/modellstudiengang-medizin/thesima/], the mandatory encounters with SPs usually take place physically in small groups. In this way, we aim to achieve the highest possible authenticity of the simulated conversation, which in our opinion makes a decisive contribution to the learning experience of students. In our view, a high degree of authenticity means both a convincing acting performance of the SPs and a natural flow of conversation on a verbal and non-verbal level as well as an intensive group dynamic and teaching atmosphere. Now, due to the COVID-19 pandemic, physical presence teaching has been cancelled and should be replaced by new teaching formats with a low risk of infection. The teaching of medical conversation was also affected by this. In the following, we will report on the specific measures taken by the Mannheim learning environment TheSiMa to enable authentic SP-based teaching despite the pandemic and discuss which conclusions were drawn for the future.

## 2. Project description

In spring 2020, SP teaching had to be adapted to the pandemic conditions in the shortest time possible. The special challenge was to afford an authentic, emotional and constructive conversation between SPs and students without physical contact. As a solution, a concept was developed in which the SPs were provided with a room in the learning environment in compliance with the hygiene regulations. They stayed alone in this room and participated in the lessons digitally via a laptop provided. For this purpose, the faculty selected the software “HeiConf” [https://heiconf.uni-heidelberg.de/], which is considered to be secure for data protection reasons. The students, in turn, connected to the lessons from home via HeiConf. From May to August 2020, 176 teaching units of 45 minutes each were conducted in this way. A total of 354 students from the academic years 3, 4 and 5 took part in the teaching units. Each lesson unit included a 10-30-minute conversation with SPs, which gave students the opportunity to apply and practice conversation techniques such as NURSE and SPIKES. After the conversations, the students received structured feedback [7] from the SPs, their fellow students and lecturers. The conversations between SPs and students were recorded. During the period of May-August 2020, 18 SPs participated in such teaching units, each playing a different role. A total of 15 different complex roles or scenarios were portrayed, such as the delivery of a cancer diagnosis, an initial conversation after a suicide attempt and a conversation about quitting smoking. After the teaching units, oral feedback about the digital teaching concept was gathered from the SPs and students.

## 3. Results

Students stated that the online teaching units on medical conversation with SPs were surprisingly similar to the learning experience with the usual classroom teaching. Thus they could use and train learned conversation techniques well in the discussion. All 18 SPs interviewed stated that the teaching units worked out better than they had expected. In addition, they did not feel restricted in their role portrayal by the digital format. In fact, when analyzing video recordings of the conversations, even the portrayal of highly emotional scenarios such as the delivery of a cancer diagnosis or the conversation after a suicide attempt seemed authentic. One point of criticism, which was mentioned by a majority of the SPs and students interviewed, related to the non-verbal aspects of the conversation as well as the group dynamics and atmosphere of the classroom. The feedback at the end of a conversation was mainly related to verbal aspects of communication and the application of the conversation techniques learned, whereas non-verbal aspects such as eye contact, posture and distance were assessed less intensively. The emotionality of the group and the intensity of the group dynamics in the discussion phase after the SP encounter fell significantly compared to the face-to-face sessions, which limited the authenticity and quality of the learning experience.

## 4. Discussion and outlook

The digital implementation of the SP-based teaching on medical conversation under pandemic conditions has been a simple and constructive way to promote the communicative skills of the students during the last months. It was stated that concrete conversation techniques as well as the observation of conversation structures can be trained well online. Therefore, this should also be done in the future within the framework of online teaching, in addition to the existing teaching model with SPs in physical presence. It is planned that future students will first learn to recognize and apply concrete conversation techniques and structures in a technically oriented online class. In subsequent physical teaching units with SPs, the focus will lay on non-verbal and atmospheric aspects of conversation as well as the emotional experience of the student group. It is planned to evaluate how such a blended learning format can improve the communicative skills of students.

## Competing interests

The authors declare that they have no competing interests. 
